# Changes in left atrial function in patients undergoing cardioversion for atrial fibrillation: relevance of left atrial strain in heart failure

**DOI:** 10.1007/s00392-021-01982-0

**Published:** 2021-12-21

**Authors:** Maximilian von Roeder, Stephan Blazek, Karl-Philipp Rommel, Karl-Patrik Kresoja, Guglielmo Gioia, Luise Mentzel, Julia Anna Lurz, Christian Besler, Karl Fengler, Gerhard Hindricks, Steffen Desch, Holger Thiele, Philipp Lurz

**Affiliations:** 1 Department of Internal Medicine/Cardiology, Heart Center Leipzig at University of Leipzig, Strümpellstr. 39, 04289 Leipzig, Germany; 2 Department of Electrophysiology, Heart Center Leipzig at University of Leipzig, Leipzig, Germany

**Keywords:** Heart failure, Preserved ejection fraction, Atrial function, Speckle-tracking, Echocardiography, Atrial fibrillation

## Abstract

**Background:**

Left atrial (LA) reservoir strain provides prognostic information in patients with and without heart failure (HF), but might be altered by atrial fibrillation (AF). The aim of the current study was to investigate changes of LA deformation in patients undergoing cardioversion (CV) for first-time diagnosis of AF.

**Methods and results:**

We performed 3D-echocardiography and strain analysis before CV (Baseline), after 25 ± 10 days (FU-1) and after 190 ± 20 days (FU-2). LA volumes, reservoir, conduit and active function were measured. In total, 51 patients were included of whom 35 were in SR at FU-1 (12 HF and preserved ejection fraction (HFpEF)), while 16 had ongoing recurrence of AF (9 HFpEF). LA maximum volume was unaffected by cardioversion (Baseline vs. FU-2: 41 ± 11 vs 40 ± 10 ml/m^2^; *p* = 0.85). Restored SR led to a significant increase in LA reservoir strain (Baseline vs FU-1: 12.9 ± 6.8 vs 24.6 ± 9.4, *p* < 0.0001), mediated by restored LA active strain (SR group Baseline vs. FU-1: 0 ± 0 vs. 12.3 ± 5.3%, *p* < 0.0001), while LA conduit strain remained unchanged (Baseline vs. FU-1: 12.9 ± 6.8 vs 13.1 ± 6.2, *p* = 0.78). Age-controlled LA active strain remained the only significant predictor of LA reservoir strain on multivariable analysis (*β* 1.2, CI 1.04–1.4, *p* < 0.0001). HFpEF patients exhibited a significant increase in LA active (8.2 ± 4.3 vs 12.2 ± 6.6%, *p* = 0.004) and reservoir strain (18.3 ± 5.7 vs. 22.8 ± 8.8, *p* = 0.04) between FU-1 and FU-2, associated with improved LV filling (*r* = 0.77, *p* = 0.005).

**Conclusion:**

Reestablished SR improves LA reservoir strain by restoring LA active strain. Despite prolonged atrial stunning following CV, preserved SR might be of hemodynamic and prognostic benefit in HFpEF.

**Graphical abstract:**

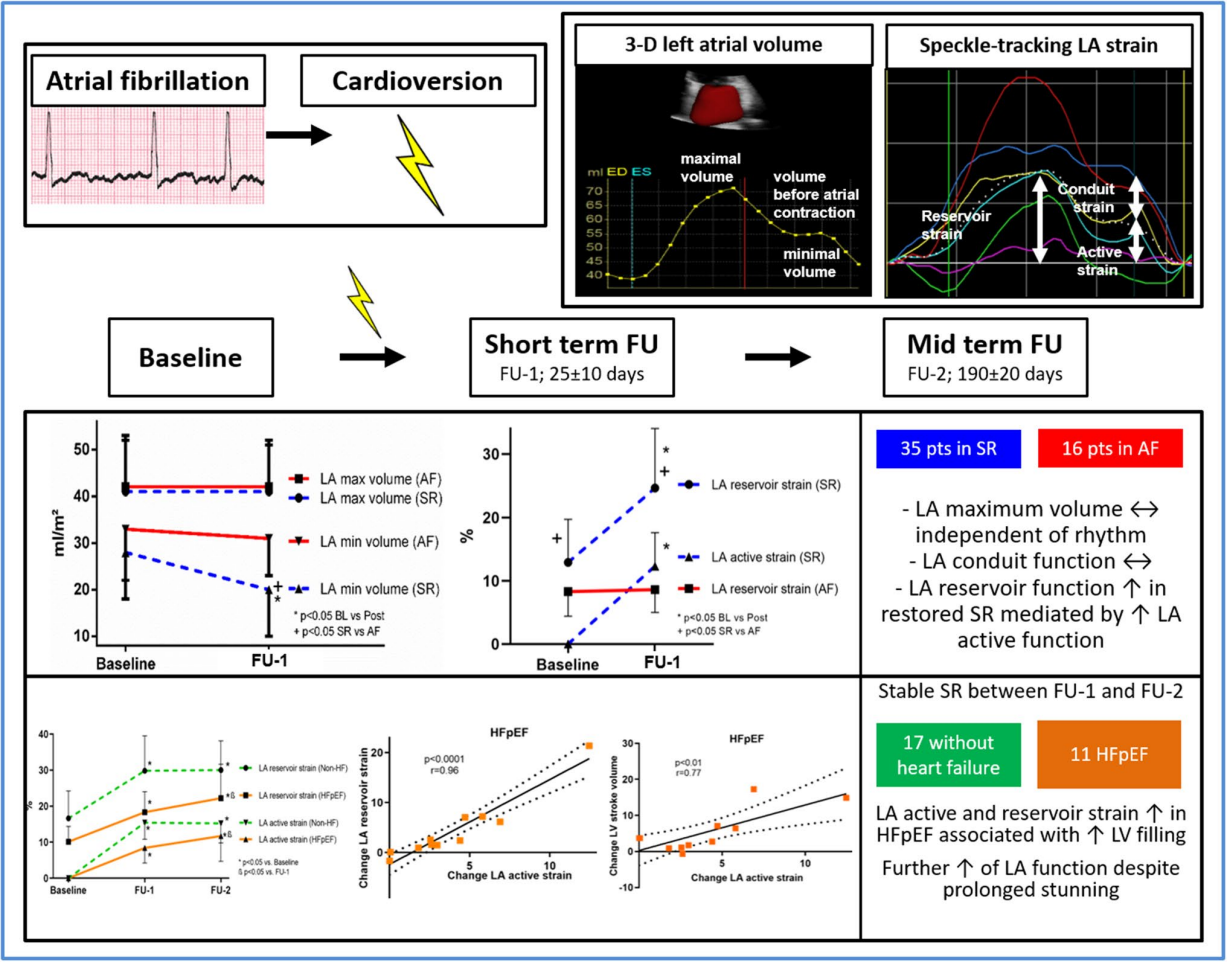

## Introduction

Left atrial (LA) reservoir strain has emerged as an important marker for diagnosis and risk prediction in patients at risk of heart failure (HF) and in patients with established HF with preserved (HFpEF), mid-range or reduced ejection fraction (HFrEF) [1–5].

Atrial fibrillation (AF) is an important aggravator of morbidity and mortality in HF [6]. Patients with AF on the other hand are at increased risk of developing HF [7]. Recently, large registry studies and meta-analyses evaluating normal values of LA reservoir, conduit and active function have been published [8, 9], but these excluded patients with AF or HF.

The influence of AF on the different aspects of LA function and the effect of altered LA deformation on left ventricular (LV) function remains to be elucidated. Cardioversion (CV) can restore sinus rhythm (SR) in patients with AF, but frequently myocardial stunning is present in patients with longer lasting AF [10]. Previous studies derived LA function from Doppler-echocardiography, which is angle-dependent, and from 2-dimensional echocardiography, which hampers the assessment of the complex LA geometry. Three-dimensional echocardiography (3-DE) allows for full volume coverage of the LA and in conjunction with speckle-tracking echocardiography (STE) for a profound analysis of LA size and function [8]. The aims of the current study were, therefore, to analyze: (1) whether STE and 3-DE derived LA function in patients with a first diagnosis of AF undergoing cardioversion is changed by successfully restoring SR, (2) whether LA function in patients with HFpEF responds in the same way as in patients without HF, and (3) if LA function is associated with LV function and clinical status.

## Methods

### Study protocol

Consecutive patients with a first diagnosis of symptomatic AF presenting to the emergency department of the Heart Center Leipzig at University of Leipzig scheduled for electrical CV on the same day were prospectively recruited. Exclusion criteria were as follows: age < 18 years, insufficient image quality on transthoracic echocardiography, previous episode of AF, hemodynamic instability, need for intensive care admission, ≥ moderate valvular regurgitation or stenosis, LVEF < 50% after CV/rate control, significant cardiomyopathy, myocardial infarction < 6 month ago, unstable angina pectoris.

Clinical examination, blood analysis (including NT-proBNP) and echocardiography were performed prior to CV (Baseline), after 2–4 weeks (short-term follow-up, FU-1) and after 6 months (mid-term follow-up, FU-2).

During the FU-1 visit, while in SR or under effective rate control (target < 110 bpm), patients were stratified into suffering from HFpEF (HFpEF cohort) or not (Non-HF cohort) according to the consensus paper of the ESC Heart Failure Association using the HFA-PEFF-score ranging from 0–6 points [11]. The groups were defined as follows: HFpEF: (1) signs and/or symptoms of heart failure and (2) a value of ≥ 5 on the HFA-PEFF-score. Non-HF: not fulfilling the criteria mentioned above.

Patients were also classified according to their response to CV/AF status as restored sinus rhythm (SR) if they were in SR at the time of FU-1/FU-2 or if they had recurrent AF (RAF). RAF patients without achieving effective rate control were excluded from the analysis. RAF was defined as recurrence of AF on a 12-lead-ECG during FU-1 or FU-2.

The study was approved by the local ethics committee of the University of Leipzig and all patients gave written informed consent.

### Echocardiography

Echocardiography was performed on a Vivid E9 (GE Healthcare, Chalfont St. Giles, Great Britain) by experienced physicians and analyzed offline using commercially available software (Echopac PC 6.1.0, GE Healthcare). LV size and function were quantified according to current guidelines [12]. Diastolic properties were assessed by determining transmitral early (E-wave) and late (A-wave) flow velocities on pulsed-wave Doppler and by corresponding tissue Doppler peak diastolic velocities of the septal and lateral mitral annulus (e′) [13]. All diastolic properties were measured during the FU-1-visit under controlled heart rate. The velocity–time-integral from pulsed-wave Doppler of the A-wave was measured in patients with SR.

### Left atrial size and function

LA volumes were measured from a focused 3D dataset covering the whole LA with a rate > 30 volumes per second. Tracking was carefully reviewed and in case of insufficient automated tracking, manual adjustments were made. Maximal and minimal LA volumes (LAV_max_, LAV_min_) were derived from the time volume curve and atrial volume pre atrial contraction (LAV_pac_) was measured at the beginning of the P-wave in case of SR [8] and in case of AF LAV_pac_ equals LAV_min_. Corresponding EFs were calculated from these volumes by dividing stroke volume (SV) by volume before contraction × 100. Atrial strain curves were derived from 2D images of the apical two- and four-chamber-view and averaged values for LA reservoir strain, LA conduit strain and LA active strain were measured as previously described and the “zero point” was set at the QRS complex [14]. The accuracy of tracking was visually confirmed and the region of interest readjusted if necessary.

**Fig. 1:**
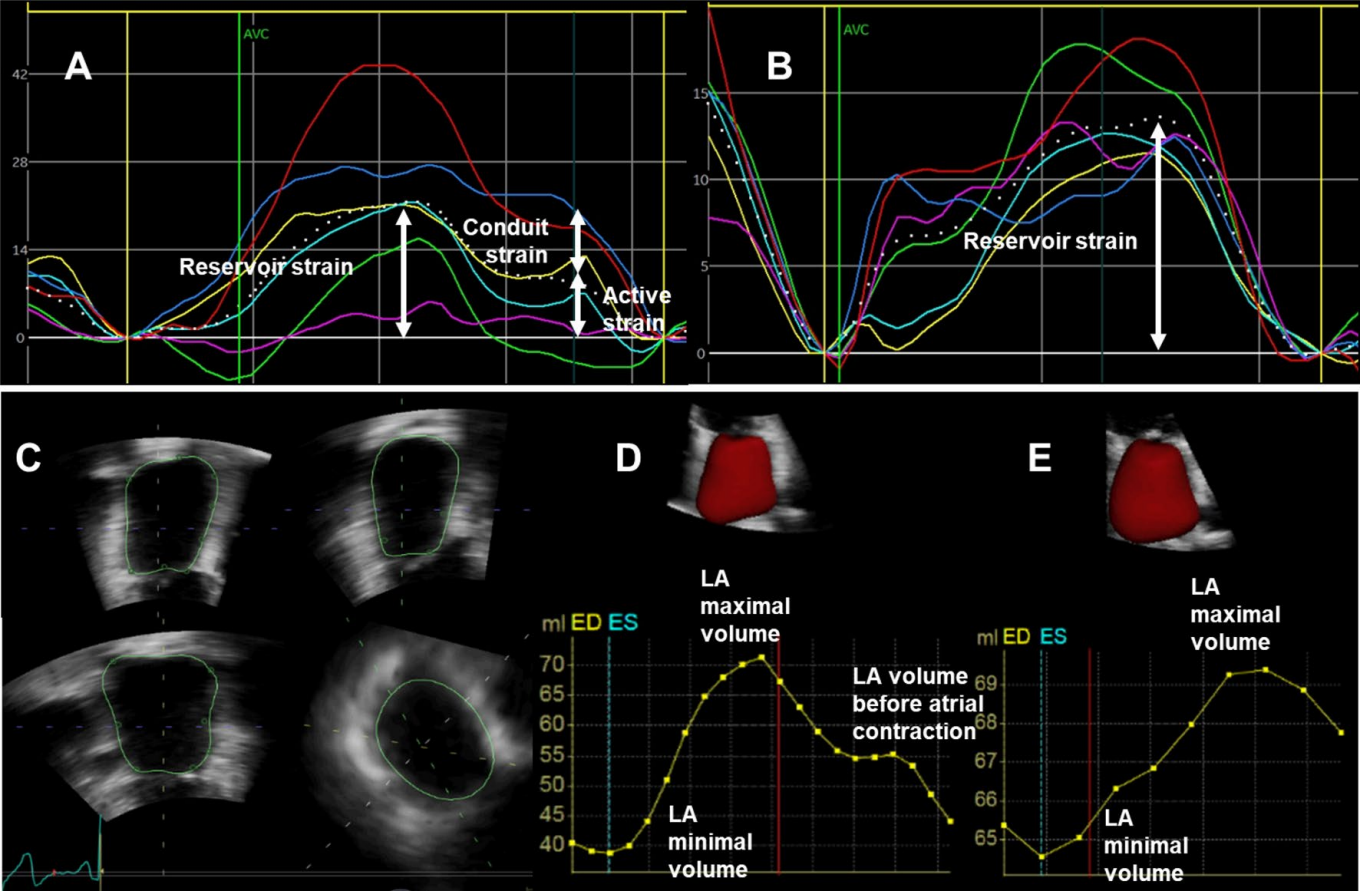
Left atrial strain curves in SR (**A**) and AF (**B**), dashed curve represents the average strain, the scale was set automatically by the program and is lower in AF, missing atrial contraction in AF (**C**): 3-D LA volume tracing, Left atrial volume curves in SR (**D**) and AF (**E**)

The following aspects of LA function were measured: LA reservoir function (LA total EF, LA reservoir strain), LA conduit function (LA conduit EF, LA conduit strain) and LA active function (LA active EF, LA active strain). Examples of LA volume and strain curves are shown in Fig. 1.

### Statistical analyses

Data for continuous variables are presented as mean ± standard deviation (SD), if normally distributed, or as median and interquartile range (IQR) if non-normally distributed. Distribution was tested using Shapiro–Wilk tests. Categorical variables are presented as frequencies and percentages. Comparisons between groups were made using Fisher’s exact tests for categorical variables. Continuous variables were compared with unpaired *t* tests or non-parametric Mann–Whitney *U* tests where appropriate. Sequential measurements were compared by repeated measures ANOVA. Atrial strain measurements are reported in absolute values even though LA conduit and active strain have negative values.

Pearson’s correlation (*r*), Spearman’s correlation (*ρ*) and linear regression were used to assess associations with LA reservoir strain. Stepwise forward multivariable linear regression analysis was performed to control for influencing factors of LA reservoir strain. Unstandardized beta coefficients (*ß*) and confidence intervals are reported for multivariable regression analysis. All data were analyzed using SPSS Version 25 (IBM, Armonk, NY, USA).

## Results

### Patient cohort

**Fig. 2:**
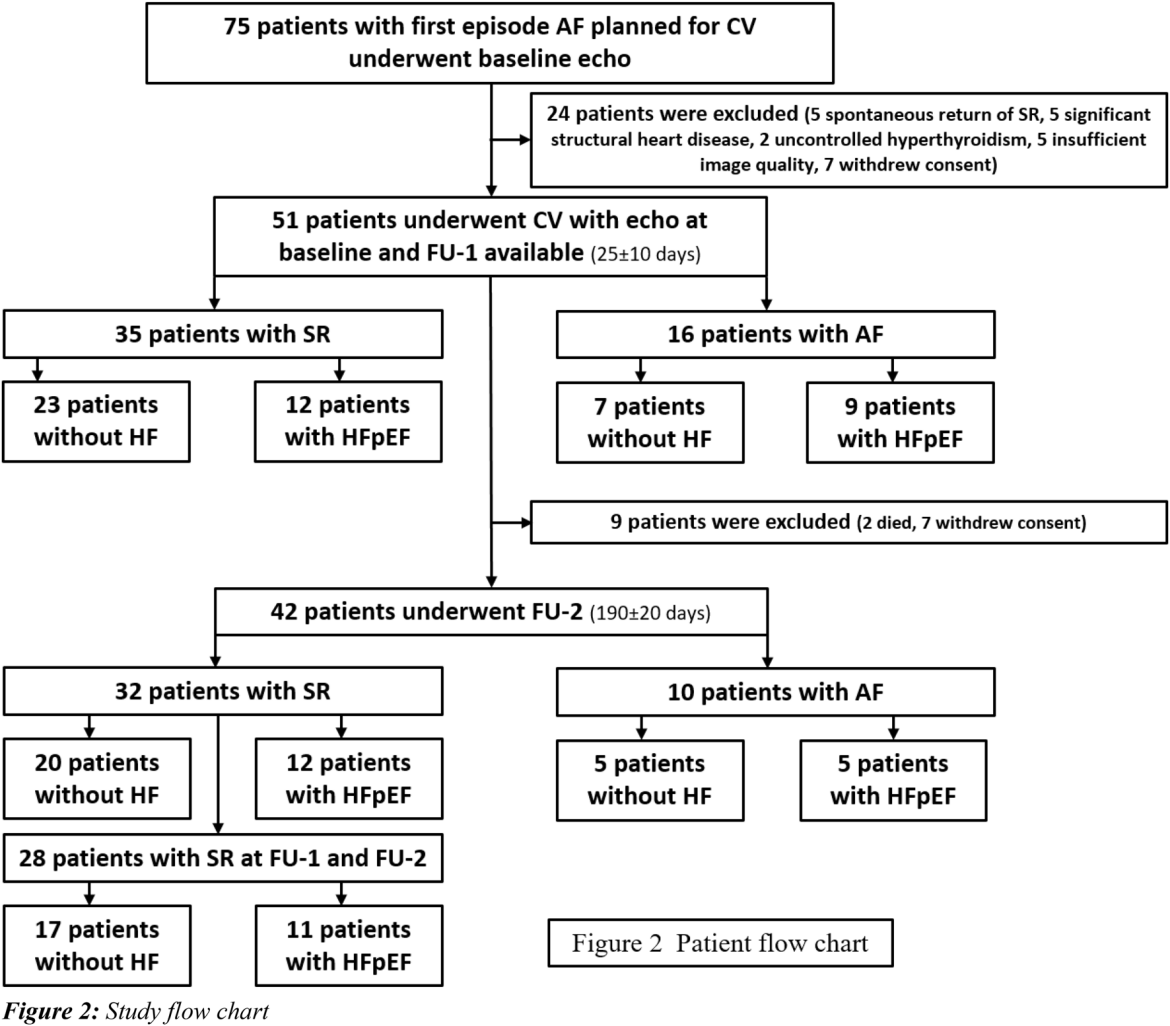
Study flow chart

Overall, 75 patients were screened for the analysis and provided written informed consent. Of these, 24 patients were excluded between Baseline and FU-1-visit (25 ± 10 days after CV) and another 9 were excluded between FU-1 and FU-2 (187 ± 11 days after CV) visit (Fig. 2), patient flow chart). CV was primarily successful in all patients, but during FU-1 only 35/51 (69%) patients were in SR, while 16/51 (31%) showed AF recurrence with adequate rate control (heart rate 86 ± 17/min). In 21 patients, the diagnosis HFpEF according to the consensus paper of the ESC Heart Failure Association was established, while 30 patients were free of heart failure (Non-HF).

In total, 42 patients were available for FU-2 analysis, of whom 32 were in SR, 10 had AF and 17/42 (40%) suffered from HFpEF. In the SR group, 5 patients were on antiarrhythmic medication during FU-2 (amiodarone in all cases, 1 patient since baseline, 4 patients since FU-1). Overall, 28 patients had stable SR during FU-1 and FU-2. At FU-2, 4 patients were in SR after initiation of amiodarone at FU-1 and 1 patient had recurrence of AF at FU-2 having presented with SR at FU-1. During the time-course of the study, no patient underwent catheter ablation for atrial fibrillation.

### Baseline characteristics

Median age of the whole cohort was 70 years (IQR 57–75) and 29% were female. HFpEF patients were older, more often female and had per definition a higher HFA-PEFF-score. Cardiovascular risk factors were well balanced between HFpEF and Non-HF patients and between patients with SR and RAF. Patients with RAF had longer symptom duration before CV than patients with successful restoration of SR (21 vs. 7 days, *p* = 0.003). Baseline characteristics are presented in Table 1.

**Table 1:** Baseline characteristics

	All (*n* = 51)	HFpEF (*n* = 21)	Non-HF (*n* = 30)	SR (*n* = 35)	RAF (*n* = 16)	*p* HFpEF vs Non-HF	*p* SR vs RAF
Age (years)	70 (IQR 57–75)	74 (IQR 71–78)	59 (IQR 51–70)	69 (IQR 54–76)	71 (IQR 63–73)	**< 0.0001**	0.86
Female sex	15/51 (29%)	12/21 (57%)	3/30 (10%)	11/35 (31%)	4/16 (25%)	**< 0.0001**	0.75
BMI (kg/m^2^)	28 ± 4	29 ± 4	27 ± 3	29 ± 3	27 ± 4	**0.02**	0.06
HFA-PEFF-score	5 (IQR 3–6)	6 (IQR 6–6)	4 (IQR 3–5)	4 (IQR 3–6)	5.5 (IQR 4.25–6)	**< 0.0001**	0.22
CHADS2-VASc-Score	2 (IQR 1–4)	3 (IQR 2–4)	1 (IQR 0–3)	2 (IQR 1–4)	2.5 (IQR 1–3)	**< 0.0001**	0.78
Symptom duration (days)	10 (IQR 3–15)	10 (IQR 6–21)	6 (IQR 1–14)	7 (IQR 2–14)	21 (IQR 6–25)	0.06	**0.003**
Heart failure	21/51 (41%)	21/21 (100%)	0/30 (0%)	12/35 (34%)	9/16 (56%)	n.a	0.14
Restored sinus rhythm at FU-1	35/51 (69%)	12/21 (57%)	23/30 (77%)	35/35 (100%)	0/16 (0%)	0.22	n.a
Heart rate (beats/min)	113 ± 23	115 ± 21	112 ± 25	115 ± 21	110 ± 28	0.73	0.51
NYHA II	19/51 (37%)	19/21 (90%)	0/30 (0%)	11/35 (31%)	8/16 (50%)	**< 0.0001**	0.33
NYHA III	2/51 (4%)	2/21 (10%)	0/30 (0%)	1/35 (3%)	1/16 (6%)		
NT-proBNP (ng/l)	1204 (IQR 570–2278)	1787 (IQR 1080–3181)	932 (IQR 356–1758)	1204 (IQR 547–2278)	1315 (IQR 591–2303)	**0.004**	0.96
Hypertension	41/51 (80%)	18/21 (86%)	23/30 (77%)	29/35 (83%)	12/16 (75%)	0.50	0.71
Diabetes mellitus	11/51 (22%)	5/21 (24%)	6/30 (20%)	8/35 (23%)	3/16 (19%)	0.75	1.0
Hypercholesterolemia	11/51 (22%)	6/21 (29%)	5/30 (17%)	6/35 (17%)	5/16 (31%)	0.33	0.29
Coronary artery disease	3/51 (6%)	3/21 (14%)	0/30 (0%)	1/35 (3%)	2/16 (13%)	0.06	0.23
Smoking	11/51 (22%)	5/21 (24%)	6/30 (20%)	8/35 (23%)	3/16 (19%)	0.74	1.0
*ß*-blockers	32/51 (63%)	16/21 (77%)	16/30 (53%)	14/35 (40%)	5/16 (31%)	0.14	0.76
ACE-inhibitors/ARB	28/51 (55%)	13/21 (62%)	15/30 (50%)	20/35 (57%)	8/16 (50%)	0.57	0.76
Other antihypertensive drugs	22/51 (43%)	6/21 (29%)	16/30 (53%)	16/35 (46%)	6/16 (38%)	0.09	0.76
Aldosterone antagonists	2/51 (4%)	2/21 (10%)	0/30 (0%)	2/35 (6%)	0/16 (0%)	0.17	1.0
Diuretics	17/52 (33%)	9/21 (43%)	8/30 (27%)	13/35 (37%)	4/16 (25%)	0.25	0.53

Values are presented as means ± standard deviation, medians + interquartile range (IQR) or frequencies (percentages)

*BMI* body mass index, *NYHA* New York Heart Association Class, *NT-proBNP* N-terminal pro-B-type natriuretic peptide, *ACE* angiotensin converting enzyme, *ARB* Angiotensin receptor blocker, *p *values below the significance level of 0.05 are highlighted in bold

Restoration of SR led to a significant decrease in NT-proBNP (Baseline vs. FU-1, 1204 (IQR 547–2278) vs. 278 (IQR 80–565) ng/l, *p* < 0.0001), while no significant changes were observed in RAF (Baseline vs. FU-1, 1315 (IQR 591–2303) vs. 1041 (IQR 780–1660) ng/l, *p* = 0.50). HFpEF patients had higher NT-proBNP levels at all time-points (e.g. FU-1 768 (IQR 424–1563) vs. 242 (IQR 61–582) ng/l, *p* < 0.0001, FU-2 472 (IQR 348–1222) vs. 123 (IQR 36–423) ng/l, *p* = 0.0003).

### Left ventricular function

Table 2 gives a summary of LV systolic and diastolic function. Despite tachyarrhythmia, Baseline average LV ejection fraction was preserved and improved in the overall cohort after SR restoration or adequate rate control (52 ± 16% vs. 60 ± 7%, *p* = 0.001). Restoration of SR led to a significant increase in LVEDV, LVEF and LVSV. In RAF, a small but significant increase in LVSV was observed. At FU-1, LVEDV, LVESV and LVSV were significantly higher in SR as compared to RAF. As a consequence of a higher heart rate (61 ± 8 vs. 86 ± 17/min, *p* = 0.0002), LV cardiac index did not differ between the SR and RAF group (2.3 ± 0.5 vs. 2.3 ± 0.4 l/min/m^2^). E/e’ was not different between restored SR and RAF.

**Table 2:** Echo characteristics

	All (*n* = 51)	HFpEF (*n* = 21)	Non-HF (*n* = 30)	SR (*n* = 35)	RAF (*n* = 16)	*p* HFpEF vs Non-HF	*p* SR vs RAF
Baseline							
LVEDV (ml/m^2^)	52 ± 16	48 ± 16	54 ± 16	55 ± 18	44 ± 10	0.21	**0.03**
LVESV (ml/m^2^)	25 ± 16	23 ± 15	27 ± 18	28 ± 19	20 ± 8	0.41	0.13
LVSV (ml/m^2^)	26 ± 8	25 ± 9	27 ± 7	27 ± 9	24 ± 5	0.37	0.12
LVEF (%)	53 ± 14	54 ± 13	53 ± 15	52 ± 16	55 ± 11	0.77	0.48
LAV_max_ (ml/m^2^)	41 ± 11	45 ± 10	38 ± 10	41 ± 11	42 ± 11	**0.02**	0.69
LAV_min_ (ml/m^2^)	30 ± 11	34 ± 10	27 ± 11	28 ± 10	33 ± 11	**0.02**	0.14
LA reservoir strain (%)	11.5 ± 6.4	8.9 ± 3.9	13.3 ± 7.2	12.9 ± 6.8	8.3 ± 3.9	**0.02**	**0.02**
FU-1							
Heart rate 1/min	69 ± 19	73 ± 21	67 ± 16	61 ± 8	86 ± 17	0.20	**0.0002**
LVEDV (ml/m^2^)	59 ± 15 *	55 ± 17 *	62 ± 13 #	64 ± 15*	47 ± 9	0.15	**< 0.0001**
LVESV (ml/m^2^)	24 ± 8	22 ± 10	25 ± 7	26 ± 8	19 ± 6	0.17	**0.007**
LVSV (ml/m^2^)	35 ± 8*	33 ± 9*	36 ± 8*	38 ± 7*	28 ± 7^#^	0.21	**< 0.0001**
LV cardiac index (l/min/m^2^)	2.3 ± 0.5	2.3 ± 0.5	2.3 ± 0.5	2.3 ± 0.5	2.3 ± 0.4	0.81	0.82
LVEF (%)	60 ± 7*	61 ± 8*	60 ± 5 #	60 ± 5	59 ± 10	0.25	0.66
Transmitral E max (m/s)	0.88 ± 0.18	0.91 ± 0.18	0.70 ± 0.19	0.74 ± 0.21	0.89 ± 0.17	**0.0002**	**0.02**
e’ average (m/s)	0.08 ± 0.02	0.07 ± 0.02	0.09 ± 0.03	0.07 ± 0.02	0.09 ± 0.02	**0.04**	0.07
E/e’	10.4 ± 4.9	13.3 ± 4.2	8.7 ± 3.5	10.5 ± 4.4	11.0 ± 4.5	**0.0001**	0.73
Systolic transtricuspid gradient (mmHg)	27.1 ± 7.0	32.4 ± 5.9	25.3 ± 4.8	28.3 ± 6.3	27.8 ± 6.4	**< 0.0001**	0.46
LV mass index elevated (♀ > 95 g/m^2^, ♂ > 115 g/m^2^; *n*, %)	9/51 (18%)	7/21 (33%)	2/30 (7%)	7/35 (20%)	2/16 (13%)	**0.03**	0.70
LAV_max_ (ml/m^2^)	41 ± 11	46 ± 10	38 ± 9	41 ± 11	42 ± 9	**0.005**	0.71
LAV_min_ (ml/m^2^)	23 ± 11*	30 ± 8 #	19 ± 10*	20 ± 10*	28 ± 10	**0.0001**	**< 0.0001**
LA reservoir strain (%)	19.6 ± 11.0*	13.3 ± 7.0*	23.9 ± 11.3*	24.6 ± 9.4*	8.5 ± 3.7	**0.0004**	**< 0.0001**
	BL vs PO *p < 0.01 ^#^*p* < 0.05						

Values are presented as means ± standard deviation or means + interquartile range

*HFpEF* Heart failure with preserved ejection fraction, *Non-HF* patients not suffering from heart failure, *SR* patients with restored sinus rhythm, *RAF* patients with recurrent atrial fibrillation, *LV* left ventricular, *EDV* end-diastolic volume, *ESV* end systolic volume, *SV* stroke volume, *EF* ejection fraction, *LA* left atrial, *V*_*max*_ maximal volume, *V*_*min*_ minimal volume

E’avg is calculated as (E’septal + E’lateral)/2, *p* values below the significance level of 0.05 are highlighted in bold

### Left atrial volume

**Fig. 3:**
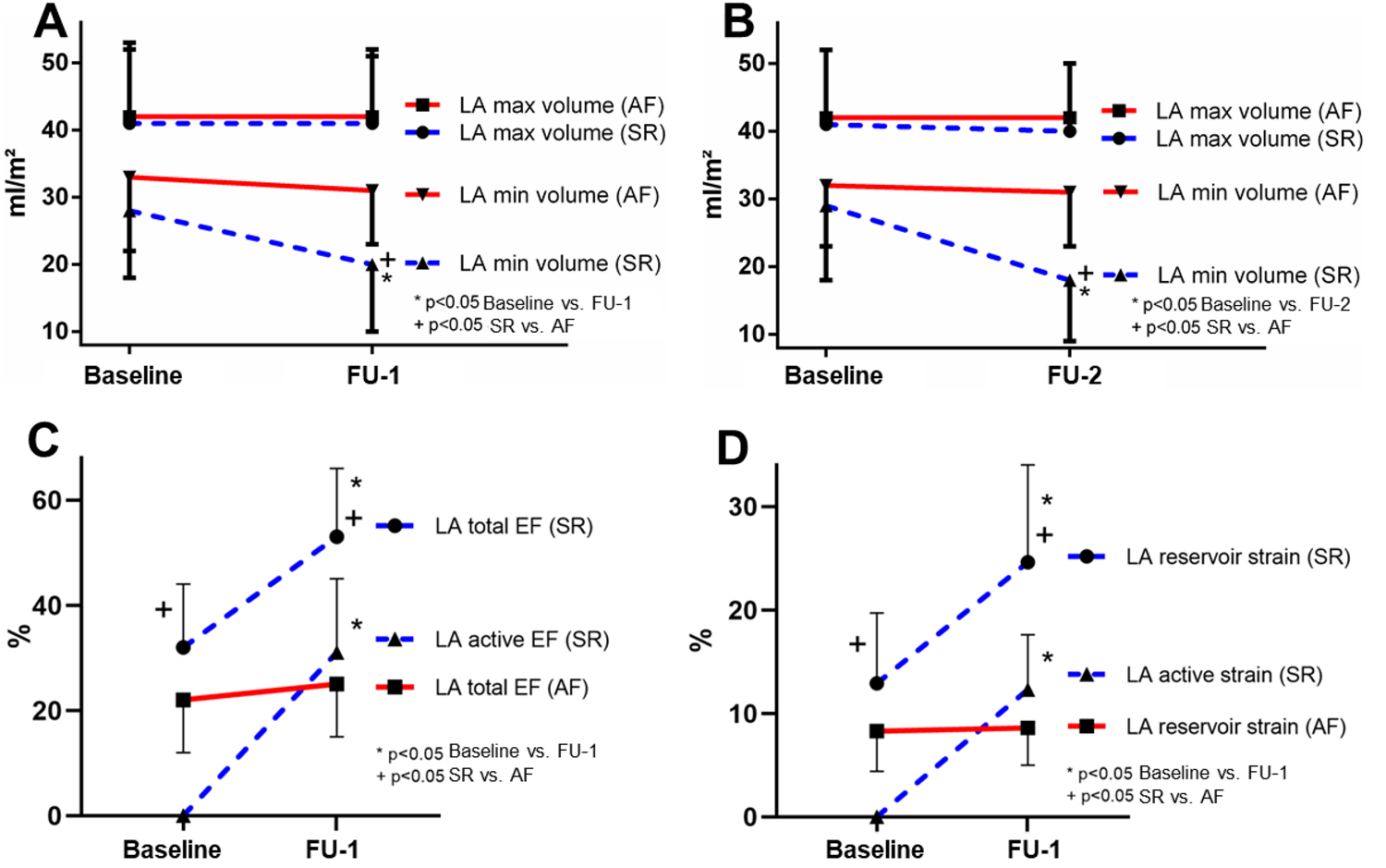
**A** LA volume change between Baseline and FU-1 (*n* = 51). **B** LA volume change between Baseline and FU-2 (*n* = 42). **C** Change in volumetric LA function between Baseline and FU-1 (25 ± 10 days after CV). **D** Change in strain-derived LA function between Baseline and FU-1 (25 ± 10 days after CV)

LAV_max_ at Baseline did not differ between patients that were converted to SR or patients that remained in AF (40 ± 10 vs. 42 ± 9 ml/m^2^, *p* = 0.63). At the FU-1-visit, LAV_max_ did not change in any cohort, but LAV_min_ decreased in patients converted to SR (Baseline vs. FU-1: 28 ± 10 vs. 20 ± 10 ml/m^2^, *p* < 0.0001), while it remained unchanged in RAF patients and was significantly higher than in SR (SR vs. RAF: 20 ± 10 vs. 31 ± 8 ml/m^2^, *p* = 0.0001). At FU-2 (187 ± 11 days), no change in LAV_max_ was observed in any group and LAV_min_ remained significantly lower in SR as compared to RAF (18 ± 9 vs. 31 ± 8, *p* = 0.0002). Figure 3, panels A + B depict the LA volume changes in SR and RAF.

### Sinus rhythm and left atrial function

At Baseline, LA reservoir function was worse in the RAF group (LA reservoir strain 8.3 ± 3.9 vs. 12.9 ± 6.8%, *p* = 0.02). Restoration of SR led to a significant increase in LA reservoir function (LA reservoir strain Baseline vs. FU-1 12.9 ± 6.8 vs. 24.6 ± 9.4, *p* < 0.0001), while LA reservoir function did not change in RAF despite a significant decrease in heart rate (LA reservoir strain Baseline vs. FU-1 8.3 ± 3.9 vs. 8.5 ± 3.7, *p* = 0.66, heart rate 110 ± 28 vs. 86 ± 17, *p* < 0.0001). LA conduit function did not change at FU-1 or FU-2 in any group. Figure 3, panels C + D show the acute changes in LA function in SR and RAF. No significant correlation between heart rate and LA reservoir strain (*r* = 0.023, *p* = 0.90) or LA conduit strain (*r* = − 0.18, *p* = 0.31) in sinus rhythm could be found.

### Importance of LA active function for LA reservoir function

LA reservoir strain correlated with LA active (*r* = 0.89, *p* < 0.0001) and conduit strain (*r* = 0.84, *r* < 0.0001). NT-proBNP (*ρ* = − 0.79, *p* < 0.0001) showed a negative correlation with LA reservoir strain. Correlations of relevant cofactors are listed in Table 3. There was no significant correlation of systolic (*r* = − 0.05, *p* = 0.71) or diastolic (*r* = 0.02, *p* = 0.88) blood pressure with LA reservoir strain. Controlling for age, LA active strain remained the only independent predictor of LA reservoir strain (β 1.2, CI 1.04–1.4, *p* < 0.0001). Considering clinical parameters only (heart rate, age, sex, HFpEF status, NT-proBNP and ongoing atrial fibrillation), a good predictive model (*r* = 0.84, *p* < 0.0001) resulted with ongoing atrial fibrillation as strongest predictor in that model (β-15, CI – 19 to − 11, *p* < 0.0001) and age as the only significant cofactor (*β* − 0.43, CI − 0.57 to − 0.29, *p* < 0.0001) while all other factors were excluded, highlighting the importance of SR restoration for improved LA reservoir strain.

**Table 3:** Uni- and multivariable predictors of LA reservoir strain

	Univariable				Multivariable		
	Correlation coefficient	Regression coefficient	Confidence interval	*p*	Regression coefficient	Confidence interval	*p*
LA active strain (%)	0.89	1.4	1.2 to 1.6	**< 0.0001**	1.22	1.04 to 1.40	**< 0.0001**
LA conduit strain (%)	0.84	1.6	1.3 to 1.9	**< 0.0001**	Not included		
Ongoing atrial fibrillation	− 0.68	− 16	− 21 to − 11	**< 0.0001**	–		
NT-proBNP (pg/ml)	− 0.65	− 0.01	− 0.1 to − 0.1	**< 0.0001**	–		
Age years	− 0.55	− 0.48	− 0.69 to − 0.27	**< 0.0001**	− 0.23	− 0.33 to − 0.12	**0.0001**
Heart rate/min	− 0.5	− 0.3	− 0.4 to 0.1	**0.0002**	-		
HFpEF	− 0.49	− 10.7	− 16.2 to − 5.2	**0.0003**	–		
Symptom duration before cardioversion days	− 0.46	− 0.6	− 0.9 to − 0.3	**0.001**	–		
E/e`	− 0.40	− 1.0	− 1.7 to − 0.3	**0.004**	–		
BMI kg/m^2^	− 0.34	− 0.97	− 1.8 to − 0.2	**0.02**	–		
LA Volume max (ml/m^2^)	− 0.29	− 0.3	− 0.6 to 0	**0.04**	–		
Female sex	0.22	–	–	0.13	–		
Baseline NT-proBNP (pg/ml)	− 0.17	–	–	0.17	–		
LV mass index elevated (♀ > 95 g/m^2^, ♂ > 115 g/m^2^)	− 0.10			0.50			
LVEF (%)	− 0.06	–	–	0.69	–		
*ß*-blocker therapy	− 0.06	–	–	0.11	–		

Uni- and multivariable correlation and regression with LA reservoir strain at FU-1, *n* = 51, Abbreviations see Table 1 + 2, Multivariable forward regression analysis included LA active strain, ongoing atrial fibrillation, NT-proBNP, age and heart rate, HFpEF status and symptom duration before cardioversion

### Influence of restored sinus rhythm on LA function in HFpEF and Non-HF

**Fig. 4:**
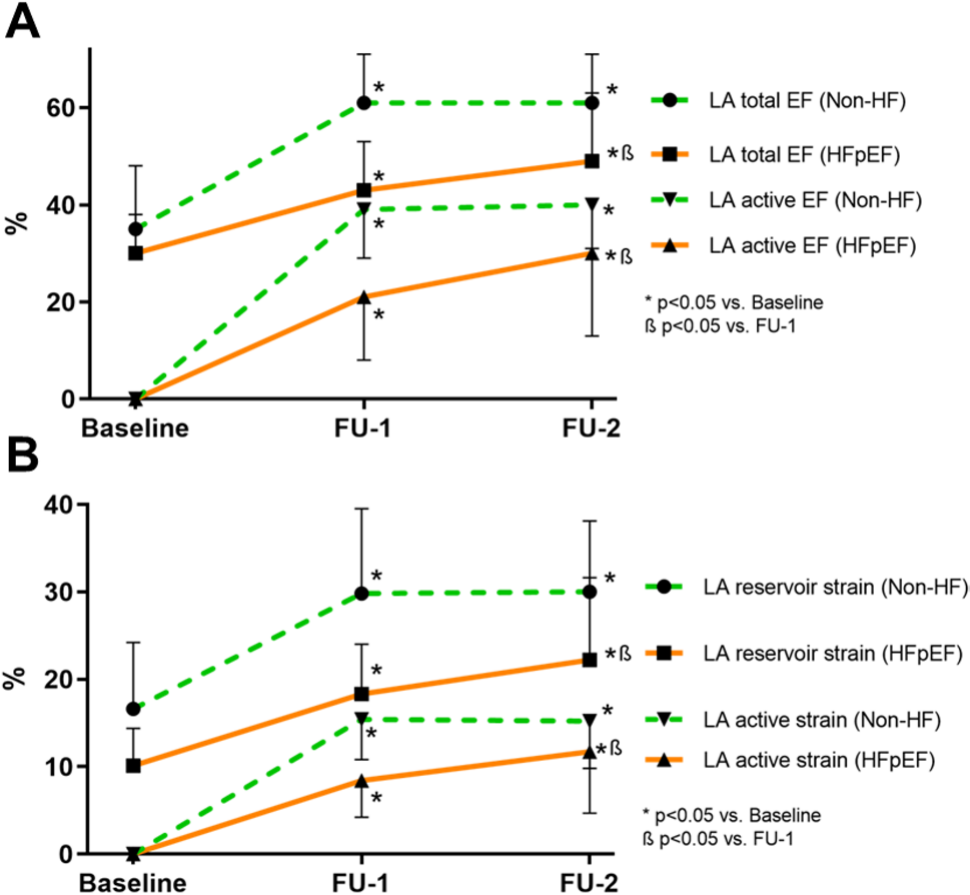
LA function in patients with stable SR throughout the course of the study (*n* = 28). Panel **A** Change in volumetric LA function, Panel **B** Change in strain-derived LA function

All aspects of LA function were lower in HFpEF as compared to Non-HF (*n* = 51, FU-1: reservoir strain 18.3 ± 5.7 vs 29.8 ± 9.7%, *p* = 0.001, conduit strain 10.2 ± 3.8 vs 15.9 ± 6.9%, *p* = 0.02 and active strain 8.2 ± 4.3 vs 15.4 ± 4.6, *p* = 0.0003). No change in any aspect of LA function was observed in Non-HF patients between FU-1 and FU-2 (*n* = 17, reservoir strain 29.8 ± 9.7 vs 30.0 ± 8.1, *p* = 0.89, LA active strain 15.4 ± 4.6 vs 15.5 ± 4.4, *p* = 0.87). However, in HFpEF patients, there was a significant increase in LA active strain between FU-1 and FU-2 (*n* = 11, 8.2 ± 4.3 vs 12.2 ± 6.6%, *p* = 0.004), which was associated with a change in LA total strain (*r* = 0.96, *p* < 0.0001 in HFpEF) and, correspondingly, LA reservoir strain increased significantly in HFpEF patients from FU-1 to FU-2 (18.3 ± 5.7 vs. 22.8 ± 8.8, *p* = 0.04) (Fig. 4). Contribution of LA active strain to LA reservoir strain remained unchanged in Non-HF (FU-1 vs FU-2: 53 ± 11 vs. 53 ± 11%, *p* = 0.81), but increased significantly in HFpEF (FU-1 vs FU-2: 44 ± 14 vs. 49 ± 15%, *p* = 0.01).

### Hemodynamic and clinical consequences of improved LA function in HFpEF

**Fig. 5:**
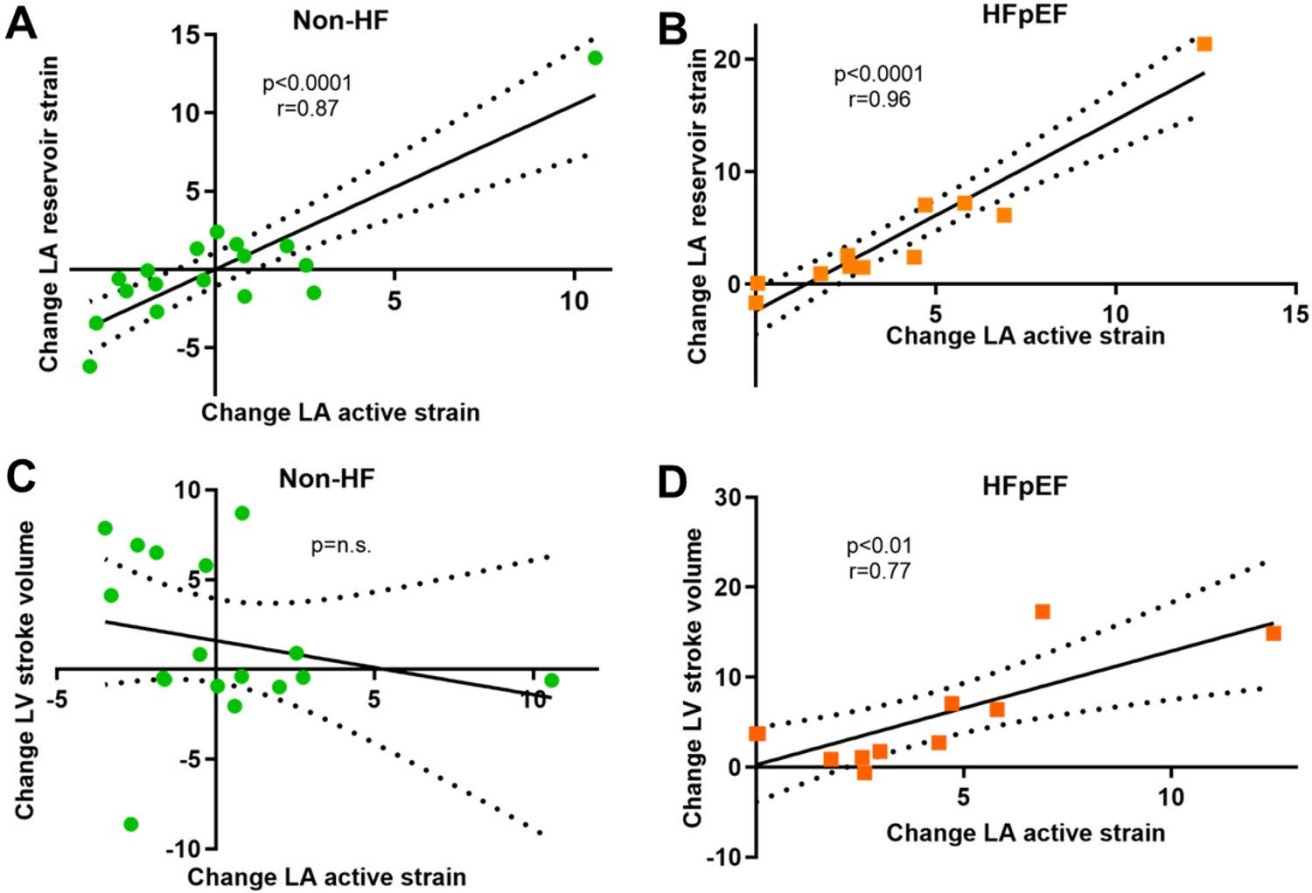
Patients with stable SR throughout the course of the study: correlation between the change in LA active strain and LA reservoir strain in Non-HF (**A**) and HFpEF (**B**) patients. Correlation between the change in LA active strain and LV stroke volume in Non-HF (**C**) and HFpEF (**D**) patients

The increased contribution of LA active strain to LA reservoir strain in HFpEF patients was associated with improved LVSV (*r* = 0.77, *p* = 0.005) and increased transmitral velocity–time-integral during A-wave increased (5.9 ± 4.7 vs. 7.3 ± 5.5 cm, *p* = 0.04), suggesting improved LV filling. Figure 5 shows the correlation between LA and LV function. NT-proBNP decreased significantly in HFpEF patients with stable SR between FU-1 and FU-2 (581 (IQR 423–768) vs. 393 (IQR 325–701) ng/l, *p* = 0.01).

## Discussion

The current study addressed the influence of AF and SR restoration on LA function in patients with a first episode of AF and the functional implications of improved LA mechanics in HFpEF and Non-HF patients using dedicated echocardiographic methods including 3-DE and STE derived strain analysis. The main findings can be summarized as follows: (1) Restoration of SR leads to a significant increase in LA reservoir function. (2) Improvement in LA reservoir function is mediated by restoration of LA active function with no change in LA conduit function. (3) HFpEF patients exhibit prolonged myocardial stunning with recuperation of LA active function beyond 25 ± 10 days after CV. (4) Improved LA function in HFpEF is associated with improved LV filling and a decrease in NT-proBNP.

### Effect of sinus rhythm restoration on left atrial function

Recently, the different aspects of atrial function were investigated in a large European survey and an international meta-analysis. The median LA reservoir strain was 39.4–42.5%, the median LA conduit strain 23.0–25.7% and the median LA active strain 16.3–17.4 [8, 9]. Correspondingly, LA active contribution to LA reservoir strain would be ~ 38–44%.

In our patients with sinus rhythm we found lower values of LA reservoir strain (24.6 ± 9.4%), conduit strain (13.2 ± 6.1%) and a higher contribution of LA active strain (12.3 ± 5.3%, contribution ~ 50%). The increase of LA reservoir function promoted by LA active function in SR seems intuitive. Interestingly, we did not find a change in LA conduit function over the study course of 6 months, neither in patients with SR nor RAF. Therefore, impaired LA conduit function in patients with a first-time diagnosis of AF might be reflecting a general underlying LA dysfunction. However, increased LA active function still allows for compensation in this early stage. Similar patterns have recently been demonstrated in patients with hypertrophic cardiomyopathy with impaired LA conduit function early in the course and impaired LA active function at more progressed states of the disease [15]. In addition, in patients with HFpEF without previous HF hospitalization increased right atrial active contraction has been shown to compensate for impaired right atrial conduit function [16]. Importantly, in patients with acute decompensated HF, LA reservoir and active strain are reduced and improve with decongestion while LA conduit strain remained unchanged—an observation again supporting the idea that LA conduit function is a preserved marker reflecting more chronic changes of LA function [17].

### Relevance of LA function for cardiac performance

During LA reservoir phase, the LA accommodates returning blood from the pulmonary circulation. During conduit and active pump phase the LA empties, providing filling for the LV. Impaired LA reservoir function limits the amount of accommodated blood at a given pressure, resulting in lower preload provided to the LV [18, 19]. Alternatively, LA pressure will rise and transduce pressure backwards to the pulmonary vasculature resulting in pulmonary congestion and/or pulmonary hypertension [20]. Both mechanisms can be detected in patients with and without HF: higher LA strain during exercise has been associated with higher SV index and cardiac output [21]. In an invasive study using exercise right-heart catheterization, impaired LA reservoir strain was associated with increased pulmonary capillary wedge and mean pulmonary artery pressure suggesting backward failure to be associated with LA dysfunction [22]. In a prior study, impaired LA function at rest in HFpEF patients was associated with reduced exercise capacity, independent of LV stiffness derived from invasively measured pressure–volume-loop analysis, [14] suggesting an independent contribution of LA dysfunction to exercise intolerance. In the current study, patients with restored SR and increased LA reservoir function had a higher LVSV, while E/e’ a surrogate for LA pressure was not different. Hence, additional LA active contraction improved LV filling at comparable levels of left ventricular end-diastolic pressure.

### Overlap of left atrial function, atrial fibrillation and heart failure

LA function, especially LA reservoir strain is able to predict the onset of HF [3, 23] and is associated with impaired outcome in patients with HFpEF [1, 4] or HFrEF [4, 24]. AF is both a predictor for the development of HFpEF and a risk factor of adverse outcome among patients with HFpEF or HFrEF [6, 7, 25]. Especially in HFpEF patients, LA cardiomyopathy with or without AF might play an independent pathophysiologic role leading to elevated LA pressure that exceeds LV end-diastolic pressure [26]. Progressive atrial fibrillation is associated with increased LA cardiomyopathy and scarring which might be reflected by impaired LA conduit function in our cohort of patients with ongoing atrial fibrillation [27]. Usually, the occurrence of risk factors (e.g. inflammation, obesity, ageing, arterial hypertension or diabetes mellitus) and consecutive LA cardiomyopathy precedes the occurrence of AF or HFpEF. Various degrees and phenotypes of LA cardiomyopathy might be present in our cohort, but baseline characteristics did not reveal differences between patients with successful restoration of SR and patients with RAF [28]. LA conduit function on the other hand was lower in HFpEF patients and in patients with ongoing AF and might prove helpful identifying patients with more severe forms of LA cardiomyopathy in lager cohorts.

Whether LA reservoir strain is an independent predictor of impaired outcome besides the detrimental effect of atrial fibrillation is difficult to assess. We found a strong influence of LA active contraction on LA reservoir strain in patients with a first-time diagnosis of atrial fibrillation and restored sinus rhythm. AF by itself is associated with higher pulmonary capillary wedge pressure [29, 30] and LA function is even more disturbed in patients with progressive AF, [31] which again translates into more elevated LA and pulmonary pressures [26, 32]. Interestingly, in a recent analysis of a large cohort of 4312 patients with acute HF, LA reservoir strain emerged as a good predictor of outcomes and outperformed traditional risk markers. However, in patients suffering from AF LA reservoir strain loses its predictive power [4]. Given the favorable hemodynamic effects of SR restoration on LA function and LV filling, a more aggressive therapy strategy regarding SR maintenance might be beneficial for patients with HFpEF.

### Limitations

The exploratory nature of the study resulted in a limited number of patients and should be regarded as hypothesis-generating. The follow-up period was 6 months only in a limited number of patients. A longer follow-up might have revealed changes in maximum LA volume and/or LA conduit function in patients with successfully restored SR. Clinical outcome assessment regarding HF was not systematically performed and might have given additional insights.

### Conclusion

LA reservoir strain is significantly enhanced by SR restoration in patients with a first-time diagnosis of atrial fibrillation, mediated by restoration of LA active function. Restored LA active function translates into improved LV filling in HFpEF patients, implying a potential hemodynamic benefit of a rhythm control strategy in the treatment of AF in these patients. Whether this translates into favorable clinical outcomes needs to be investigated in a clinical trial.

## Data Availability

Upon reasonable request.

## Abbreviations

3-DE: 3-Dimensional echocardiography
AF: Atrial fibrillation
RAF: Recurrent atrial fibrillation
CV: Cardioversion
EDV: End-diastolic volume
EF: Ejection fraction
ESV: End-systolic volume
HF: Heart failure
HFpEF: Heart failure with preserved ejection fraction
HFrEF: Heart failure with reduced ejection fraction
IQR: Interquartile range
LA: Left atrial
LAV_max_: Maximal left atrial volume
LAV_min_: Minimal left atrial volume
LAV_pac_: Left atrial volume pre atrial contraction
LV: Left ventricular
SD: Standard deviation
STE: Speckle-tracking echocardiography
SR: Sinus rhythm
SV: Stroke volume
